# Toward open sharing of task-based fMRI data: the OpenfMRI project

**DOI:** 10.3389/fninf.2013.00012

**Published:** 2013-07-08

**Authors:** Russell A. Poldrack, Deanna M. Barch, Jason P. Mitchell, Tor D. Wager, Anthony D. Wagner, Joseph T. Devlin, Chad Cumba, Oluwasanmi Koyejo, Michael P. Milham

**Affiliations:** ^1^Imaging Research Center, University of TexasAustin, TX, USA; ^2^Department of Psychology, Washington UniversitySt. Louis, MO, USA; ^3^Department of Psychology, Harvard UniversityCambridge, MA, USA; ^4^Department of Psychology, University of ColoradoBoulder, CO, USA; ^5^Department of Psychology and Neurosciences Program, Stanford UniversityStanford, CA, USA; ^6^Cognitive, Perceptual and Brain Sciences, University College LondonLondon, UK; ^7^Electrical and Computer Engineering, University of TexasAustin, TX, USA; ^8^Center for the Developing Brain, Child Mind InstituteNew York, NY, USA

**Keywords:** informatics, data sharing, metadata, multivariate, classification

## Abstract

The large-scale sharing of task-based functional neuroimaging data has the potential to allow novel insights into the organization of mental function in the brain, but the field of neuroimaging has lagged behind other areas of bioscience in the development of data sharing resources. This paper describes the OpenFMRI project (accessible online at http://www.openfmri.org), which aims to provide the neuroimaging community with a resource to support open sharing of task-based fMRI studies. We describe the motivation behind the project, focusing particularly on how this project addresses some of the well-known challenges to sharing of task-based fMRI data. Results from a preliminary analysis of the current database are presented, which demonstrate the ability to classify between task contrasts with high generalization accuracy across subjects, and the ability to identify individual subjects from their activation maps with moderately high accuracy. Clustering analyses show that the similarity relations between statistical maps have a somewhat orderly relation to the mental functions engaged by the relevant tasks. These results highlight the potential of the project to support large-scale multivariate analyses of the relation between mental processes and brain function.

## 1. Introduction

The sharing of data has become commonplace in many parts of science, and the availability of large databases of shared data has led to impressive advances that could not have been made without such sharing. For example, the GenBank database (http://www.ncbi.nlm.nih.gov/genbank/) contains all publicly available DNA sequences, which currently number more than 100 million annotated sequences. Using these data, a large number of data mining tools have been developed that allow computational gene discovery (i.e., mapping from sequences to specific genes) as well as prediction of the proteins that are encoded by a sequence. Such tools have greatly increased the power of molecular biology and genomics research. An excellent example of the power of these tools comes from the outbreak of *E. coli* O104:H4 infection in Germany in 2011. The genetic sequences obtained from these organisms were made public on the Internet, and within days researchers around the world had determined the genes responsible for the especially high virulence of the bacterium as well as its relation to other known *E. coli* strains. Such applications highlight one of the most important benefits of data sharing: By combining shared data into large databases, it is possible to identify relationships between effects at different levels of analysis (e.g., genetic sequence and bacterial virulence) that otherwise would be much more difficult to identify.

The open sharing of fMRI data has the potential to revolutionize cognitive neuroscience in much the same way (Van Horn and Gazzaniga, 2002; Poline et al., 2012). First, doing so would allow investigators to search for similar patterns of activity in multiple datasets, and thus to identify relations between cognitive tasks that result in these similarities. This could help address the common problem of reverse inference (Poldrack, 2006), wherein patterns of activation are informally used to infer putative mental function. The sharing of fMRI data would allow researchers to more formally assess the specificity of observed brain activity with various cognitive tasks, thereby permitting probabilistic inferences about the role of various brain regions or networks in mental function. Second, by allowing researchers to decompose the mental processes involved in each study and then test for associations between these processes and brain activity, large databases would support more direct identification of relations between mental processes and brain networks, rather than relying on associations with activation on single tasks (Poldrack et al., 2009; Yarkoni et al., 2011). Third, by making published datasets available to a wide range of researchers, open sharing would encourage the re-analysis of existing data with new analysis methods (e.g., Greicius et al., 2004). Doing so would not only obviate the need for additional data collection in some cases, but would also allow more direct comparison between previous and new analysis methods. In addition to these judicious effects on scientific knowledge, the availability of a large database of published datasets would also have a powerful impact on education and training, as new trainees and individuals at institutions without imaging resources would have access to extensive datasets. Finally, there is an ethical argument to be made that sharing of data is essential in order to fully respect the contributions of the human subjects who participate in research studies (Brakewood and Poldrack, 2013).

### 1.1. Challenges of fMRI data sharing

Although the benefits of sharing fMRI data are clear, the challenges of doing so are even clearer. The sharing of fMRI data is made difficult by a number of factors including large datasets, need for common data formats, complex metadata, and social factors.

#### 1.1.1. Large datasets

The usual fMRI dataset comprises a set of functional images (usually 4–8 scanning runs lasting 6–10 min each) along with structural brain images and other associated measurements (such as physiological and behavioral data). The functional data typically consist of 4-dimensional data sets (3 spatial dimensions x time); depending upon the number and length of scanning runs, spatial resolution, and the number of slices acquired, the raw functional data for a single subject in an fMRI study can range in size from 50 MB to more than 1 GB, and most studies have at least 15 subjects. Datasets of this size require substantial resources for storage and processing, although improvements in computing technology have made it feasible to store and process such datasets on commodity hardware. In addition, cloud-based resources make the sharing and analysis of very large datasets possible without purchasing any physical hardware.

#### 1.1.2. The need for common data formats

Ten years ago, the field of neuroimaging was a virtual Tower of Babel, with a number of incompatible image data formats used across different software packages and scanner platforms. This made early efforts at data sharing very difficult. In recent years, the field has gravitated toward two standard formats for data storage which have addressed this problem to some degree. Most MRI scanners now save the raw MRI data to the DICOM format. However, DICOM is not convenient for everyday analysis due to the fact that it requires a large number of small files. In addition, the DICOM standard varies between implementations across different scanners. Within the neuroimaging community, a standard known as NIfTI (http://nifti.nimh.nih.gov/nifti-1/) has been widely adopted in the field and is now supported by every major fMRI analysis package. The NIfTI format provides support for 3D and 4D images and supports rich metadata including orientation information, which can help alleviate problems with left-right orientation that were common in early days of fMRI. Although the NIfTI format is a step forward, differences in its implementation remain between software packages, such that problems can still arise when using data processed across multiple packages.

#### 1.1.3. Complex metadata

To describe a fMRI study fully, researchers must specify a large number of details. These include:

- MRI acquisition parameters
- Design and timing of the experimental task
- Description of the participants
- Data preprocessing and analysis procedures
- Description of the mental processes being examined
- Description of behavioral data during task performance

Recent work has begun to develop frameworks for minimal information regarding fMRI studies (Poldrack et al., 2008) and for more detailed descriptions of cognitive tasks (Turner and Laird, 2012). However, a systematic framework for describing these metadata does not yet exist, and fully describing an fMRI study thus remains a significant challenge.

#### 1.1.4. Researcher participation

Successful data sharing requires researchers who are not just willing but motivated to share their data. However, there are a number of reasons why investigators might not wish to share their data. First, data sharing requires significant effort on behalf of an investigator, and the perceived benefits have often not been sufficient to motivate this extra work [though the move toward “data papers” could help by providing published credit for data sharing; cf. Gorgolewski et al. (2013a)]. Second is the desire for exclusive rights to re-analyze the data in the future, either to test different hypotheses or to apply different analysis techniques. This is particularly the case with high-value datasets (e.g., data from special populations), where keeping the dataset private can provide a significant competitive advantage. Others might be reluctant to share data due to fear of subsequent analyses that could uncover problems with the data or invalidate the results from their publications. A recent study provided direct evidence that concerns about followup analyses may underlie the unwillingness to share; an analysis of psychology papers for which data were shared upon request vs. those that were not shared found that papers for which data were not shared had a higher rate of apparent errors in statistical reporting as well as having smaller effect sizes on average (Wicherts et al., 2011).

### 1.2. Previous fMRI data sharing projects

The first effort to openly share fMRI data was the fMRI Data Center (fMRIDC) (Van Horn et al., 2001), which was originated at Dartmouth and subsequently moved to Santa Barbara in 2007. Van Horn and Gazzaniga (2012) recently outlined the history of the project and discussed its impact and the lessons learned in the project. The fMRIDC amassed 107 fMRI datasets which remain available for shipment via physical media. Data obtained from the fMRIDC were used in at least ten papers that presented novel analyses, utilizing both single datasets as well as mega-analyses combining multiple datasets (see Van Horn and Ishai, 2007). These ranged from analyses of task-related connectivity (Mechelli et al., 2003) to one of the earliest studies of the “default mode” in Alzheimer's disease (Greicius et al., 2004) to an exploration of consciousness that combined data across multiple studies (Lloyd, 2002). The fMRIDC also aroused controversy within the neuroimaging community early in its existence when it was announced that some journals (including the *Journal of Cognitive Neuroscience* and *Proceedings of the National Academy of Sciences*) would require authors to submit their data to the center (Editorial, 2000). This reluctance of the community to participate in data sharing via fMRIDC was likely due to a number of factors including a lack of social consensus at the time regarding the value of data sharing as well as concern about the significant amount of effort required to submit datasets to the fMRIDC. Ultimately the project discontinued addition of new datasets due to a lack of continued funding, but the data remain available and it clearly played a role in establishing the utility of sharing complete fMRI data sets. The fMRIDC stands as a very important guiding example for data sharing in neuroimaging.

A more recent project has focused on open sharing of resting state fMRI data. Originally known as the *1000 Functional Connectomes Project* (FCP), and now as the International Neuroimaging Data-sharing Initiative (INDI) (Mennes et al., 2012), this project has already shared nearly 5000 subjects' worth of resting state fMRI data collected from centers around the world, making the data openly available via the web. Initial mega-analysis (i.e., reanalysis of the full combined dataset, as opposed to meta-analysis of summary statistics) of this dataset provided novel insights into the stability and variability of resting state networks (Biswal et al., 2010), and other groups have already used the data to make new discoveries about the organization of resting brain networks (Tomasi and Volkow, 2010). A limitation of the initial FCP dataset was that there was very little phenotype data included other than sex and age; however, more recently this group has begun prospectively sharing data with a greater amount of phenotype information, including the deeply-phenotyped NKI-Rockland sample (Nooner et al., 2012).

The FCP/INDI project shows how a community effort can result in the availability of large, freely-available datasets that can be used to enable novel scientific discoveries. The success of FCP/INDI also suggests that the neuroimaging community has a greater appreciation for the benefits of data sharing than it did when the fMRIDC first began 10 years ago. At the same time, it is important to highlight that by focusing on resting state fMRI, the FCP/INDI project sidesteps many of the difficult metadata problems that are present for task fMRI (in particular, the need to represent task paradigms and behavioral data in a systematic way).

In addition to these efforts at sharing raw data, another set of efforts has focused on sharing of highly processed data, namely the activation coordinates reported in papers. These include Brainmap (http://www.brainmap.org) (Laird et al., 2005), SumsDB (http://sumsdb.wustl.edu/sums/), and Neurosynth (http://www.neurosynth.org) (Yarkoni et al., 2011), each of which provides tools to perform coordinate-based meta-analyses. This approach has been very powerful, but at the same time is clearly limited by the coarseness of the data at every level; the shortcomings of coordinate-based meta-analysis in comparison to meta-analysis based on full image data have been shown by Salimi-Khorshidi et al. (2009). These results suggest that in addition to the sharing of raw data, there is likely utility in the sharing of processed data (e.g., statistical images).

## 2. The Openfmri Project

Here, we describe a new resource for the open dissemination of functional neuroimaging data, called the OpenfMRI Project (accessible online at http://www.openfmri.org). The goal of the project is to support the free and open distribution of both raw and processed neuroimaging datasets, focused primarily on whole-brain datasets from task-based fMRI studies. The project aims to use what was learned in previous data sharing efforts and take advantage of subsequent improvements in computing and information technology as well as changes in the social landscape that have made open data sharing more viable.

Some lessons learned from previous data sharing projects (such as fMRIDC and FCP/INDI) include:

- Data sharing can and should emerge from a community agreement regarding its benefits.
- The metadata required for sharing should be tailored to the specific research goals, rather than aiming for a complete representation of all possible variables of potential interest.
- Data should strictly adhere to a common organizational scheme, so that researchers can reanalyze very large datasets in a straightforward manner using automated means.
- Data should be instantly accessible over the internet, with minimal restrictions on access (except where necessary, e.g., for reasons of subject confidentiality).

### 2.1. Requirements for inclusion

One of the major goals of the OpenfMRI project is to enable whole-brain meta-analyses. For this reason, a dataset must include task-based fMRI data with coverage of the whole brain in order to be included in the OpenFMRI database; missing data at the edges of the volume can be accommodated, but datasets including coverage of only a portion of the brain will not be included (similar to the inclusion requirements for the BrainMap database). In addition, a high-resolution structural scan is necessary for each individual; additional structural scans, such as an in-plane structural image or diffusion-weighted images, are welcome if available but are not required. Finally, the metadata necessary to perform a standard statistical analysis (i.e., event onset times and durations for each experimental condition) are required. In cases where trial-by-trial behavioral data is necessary to perform the primary analysis of interest then those data are required; in other cases the submission of behavioral data is encouraged but not required.

### 2.2. Data organization

**Figure 1:**
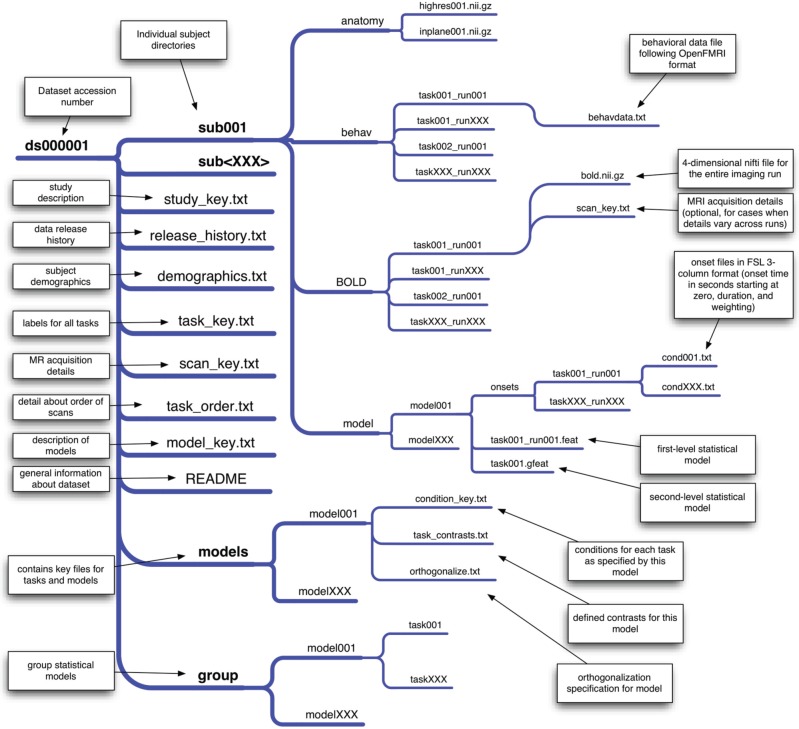
**An overview of the draft data organization scheme for the OpenFMRI project**. A schematic of the directory tree for a dataset is presented, with each subdirectory shown on a separate branch. This structure allows specification of an arbitrary set of tasks, runs, and statistical models. The key files included in the base directory for the dataset specify features that are consistent across all of the data (such as demographics, task naming, and scan ordering), while key files in subdirectories specify details that may change across models or runs.

Precise organization and naming is necessary to allow automated processing of large datasets. We have developed an initial scheme for data organization, based on the framework in use in a number of laboratories. The scheme is described in some detail by Poldrack et al. (2011b) and an overview of the current version is shown in Figure 1. The datasets currently available on the site have been organized according to this scheme. As shown in Figure 1, each study is also associated with a set of key files that describe the conditions, tasks, contrasts, and MRI data acquisition details (including order of scans) that are necessary for proper analysis. This scheme will likely need to be modified to accommodate unexpected features of future data sets, such as different types of task designs. In addition, while currently organized using flat text files, we envision that in the future this scheme will be migrated toward a more formal metadata representation scheme such as XCEDE (Gadde et al., 2012).

### 2.3. Representing fMRI designs

Probably the single most difficult challenge of sharing task-based fMRI data is the representation of metadata describing the study. A common complaint about the process of sharing data via the fMRIDC was the requirement to formally specify a very extensive body of metadata. Whereas the fMRIDC process embodied a completist philosophy about metadata, we have chosen a more minimalist approach. In particular, the metadata that we absolutely require for submission are only those metadata that are necessary for specifying the analysis of the fMRI data using standard software packages. This includes minimal details regarding the MR acquisition (e.g., the repetition time), along with a specification of the onset times and event durations for each experimental condition (which may include behaviorally-defined conditions as well as experimenter-defined conditions). In particular, we will use the flexible 3-column onset file format developed within the FSL software package; because of the flexibility of this format (which includes onset times, lengths, and weightings for each event), it is also possible to specify many different complex designs, from simple blocked designs to complex parametric event-related designs. It is also relatively easy to transform design specifications from other software packages (e.g., SPM, AFNI) into this format. When stimuli are available, they will be included either in the behavioral data file described below (e.g., for single word stimuli) or within a separate directory (e.g., for image or sound files). Each dataset will also be accompanied by a textual description of the methods (usually the methods section from an associated paper or an equivalent description for unpublished data), so that additional details can be obtained from that description even if they are not represented in the dataset. In addition, whenever DICOM header information or other detailed MRI acquisition information (e.g., a dump of the scanner protocol) is available for a study, it will be included for each scan.

### 2.4. Behavioral data

Another challenging issue surrounds the representation of behavioral data, which are essential to the modeling of fMRI data for many studies (e.g., for modeling of accuracy or reaction times). Because there is no general framework for the representation of behavioral data, we have developed a simple protocol for behavioral data storage for the OpenFMRI project. This is a trial-based scheme in which any number of variables can be specified, including independent variables (such as condition names or stimulus identities) and dependent variables (such as response time or accuracy). An additional key file describes the meaning and possible values for each variable. If additional variables need to be represented in a way that is not trial-based (e.g., eye position measured at every timepoint), these data can be specified in additional files. In this way, we allow maximal flexibility with minimal need for reformatting (since the data for most studies will already be stored in a trial-based manner within a spreadsheet). As the project progresses, we plan to develop a more formal representation of the behavioral data (e.g., using XML).

### 2.5. Psychological constructs

A final challenge arises from the need to specify the psychological constructs that are meant to be indexed by each experimental comparison in a dataset. This is a much more difficult undertaking than describing the task and imaging metadata because of the lack of common agreement about what psychological constructs are measured by any particular comparison. In a separate project known as the Cognitive Atlas [http://www.cognitiveatlas.org; Poldrack et al. (2011a)], we have begun to develop an online knowledge base (or *ontology*) that aims to capture the structure of mental processes and their relation to specific tasks. The Cognitive Atlas currently provides the basis for annotation of datasets within the OpenfMRI database; tasks included in the OpenFMRI dataset are automatically linked to the Cognitive Atlas task database, and relations between these tasks and mental processes can then be specified by researchers in the community.

## 3. Confidentiality

Confidentiality of research participants is of critical importance in data sharing (cf. Van Horn et al., 2001; Nooner et al., 2012). The upload policy for the project specifies that data should be anonymized before uploading by removing all of the 18 possible unique identifiers specified by HIPAA. The investigators submitting data are responsible for anonymization, but once data are uploaded a curator will doublecheck the data to ensure that no identifying information remains. Because high-resolution structural images may contain information about facial structures, all structural images will have facial features removed prior to sharing.

## 4. Intellectual property and credit

Another common concern about data sharing for researchers relates to intellectual property. We believe that sharing of data with highly restrictive terms and conditions would defeat the purpose of an open data sharing repository, and we trust that the community will largely be responsible in their use of the data and attribution of its provenance. For this reason, data shared by the project will be released by default under the Public Domain Dedication and License developed by the Open Data Commons (ODC). This license states that users can download the data and use them for any purpose they wish, with no requirement for permission, citation, or coauthorship. We will encourage users to follow the ODC Attribution/Share-Alike Community Norms, which request that users give credit to the originator of the data and share any resulting products in a similar manner. We realize that some investigators may wish to share high-value datasets but may not be comfortable with public domain dedication; in this case we will consider more restrictive licensing on a case-by-case basis. In cases where investigators wish to stage a dataset for release on a specific date (e.g., to coincide with the publication of a paper), we will allow investigators to specify an embargo period for submitted datasets (generally not to exceed 6 months), which will provide sufficient curation time for the dataset to be ready for release on the intended date.

Individuals sharing data should reasonably expect to receive credit for having gone to the effort of data sharing. On the OpenFMRI web site, credit is given via a link to the publication on the associated data page as well as a list of investigators involved in collecting the data. In addition, the inclusion of the OpenFMRI database within the Neuroscience Information Framework (NIF: Gardner et al., 2008) allows links to the dataset to be added automatically to the PubMed listing for each associated paper, using the NIF Link-Out Broker (Marenco et al., 2008). With the advent of venues for data publication including *Nature Scientific Data* and *GigaScience*, it is also possible for contributors to publish a separate paper that describes the dataset (for a recent example, see Gorgolewski et al., 2013b).

## 5. Processing stream

We have implemented an automated processing stream for the data in the OpenFMRI database; the processing steps are listed in Table 1, and the code for these analyses is freely available via the OpenFMRI web site. Because of the use of a precise organizational scheme and metadata format, it is possible to completely automate every step of data processing, including the generation of FSL design files for each level of analysis. Eventually, the results from each intermediate processing step will be made available in the future through the OpenFMRI web site along with the raw data. Because of the computationally intensive nature of such processing on a large dataset, analysis is performed using the high-performance Lonestar cluster at the Texas Advanced Computing Center. All code used to implement this processing stream is available at http://github.com/poldrack/openfmri.

**Table 1:** **List of processing steps applied to data, and tools used for each operation**.

**Operation**	**Tool used**
Motion correction	mcflirt (FSL)
Brain extraction (highres)	Freesurfer
Brain extraction (BOLD)	bet (FSL)
Quality assurance and generation of confound files	fmriqa (custom)
Creation of design files	custom code
First-level (within-run) statistical modeling	feat (FSL)
Second-level statistical modeling (for multi-run datasets)	feat (FSL)
Group statistical modeling	feat (FSL)
Cortical surface generation and parcellation (highres)	Freesurfer

The specific processing stream was selected based on its current use in the first author's laboratory, but represents a fairly standard processing stream in the field. After conversion to NIfTI format and organization via the standard data scheme, the BOLD data are motion-corrected using MCFLIRT (FSL) and the brain is extracted using BET (FSL). The event onsets for each experimental condition are represented using the 3-column (onset time, length, and weighting) format from FSL. Using custom code, we automatically generate the FSL design files from these onset files, with extracted motion parameters and their temporal derivatives included as nuisance regressors. First-level statistical modeling is performed using FEAT (FSL) and contrasts are automatically generated for each experimental condition compared to baseline, in addition to any other potential contrasts of interest. For studies with multiple runs per task, second-level modeling is performed using a fixed-effects model. Third-level modeling is performed using FLAME (FSL), implementing a mixed-model that treats subjects as a random effect.

High-resolution anatomical images are first brain-extracted using FreeSurfer. The anatomical image is aligned to the MNI152 template using a combination of boundary-based registration and linear registration with FNIRT (FSL). The functional images are aligned to the high resolution image and the warps are combined to provide a transformation of the functional data into the MNI152 space, which is applied to the results of the statistical analysis at the higher levels. Cortical surface generation and automated anatomical parcellation are performed using FreeSurfer.

Quality control is performed using the fmriqa package (https://github.com/poldrack/fmriqa) for raw data, and the fsl-qa package (https://github.com/poldrack/fsl-qa) for analyzed data. QA results for the raw data are included in the base download. Reports are also generated that allow manual inspection of defacing, spatial registration of structural and functional images to standard space, and statistical analyses; these reports are examined and validated by the OpenFMRI staff before the data are made publicly available.

When data are uploaded to the OpenFMRI database, they are processed by the curators through the level of group analysis, in an attempt to replicate the results of the original analysis (e.g., in a published paper associated with the dataset). Given the multiplicity of different analysis streams and likelihood of different results between streams (Carp, 2012), it is expected that the results will sometimes fail to exactly match those of the original analysis. In such a case, we first contact the investigators to ensure that the task has been properly modeled in our analysis (e.g., that there are no mistakes in the event timing files). If the modeling is confirmed to be correct, then the authors will be given a chance to withdraw their submission, or to have the data shared despite this mismatch in results.

### 5.1. Data versioning and software updates

The web page for each dataset currently contains versioning information that describes any changes in the dataset. In addition, a revision history file is included with each dataset download. While the raw data are largely independent of any processing software, the distribution of processed data is made challenging by the constant stream of software updates for packages such as FSL. Fortunately, the implementation of our processing stream within a high performance computing environment makes it relatively straightforward to reprocess the entire database within a relatively short time (generally within 1 day). For existing processed data, we will reprocess the data and release updated versions of the data for all major revisions of the FSL package, once they have been vetted and ensured to work properly with our processing stream. We do not expect substantial changes in results across major versions of the software, but if any such differences are noticed, we will first discuss with the software developers to ensure that they do not reflect software problems. If they are determined to be true methodological differences, then these differences will be described on the web site.

### 5.2. Informatics platform

The OpenFMRI website storage and processing mechanisms have been chosen to provide an extensible software platform. Datasets are stored in an XNAT server (Marcus et al., 2007), and processing streams access the datasets through XNAT's built in REST API. In our initial model, the Lonestar cluster at TACC accesses data from XNAT, performs its processing operation, and then writes the processed data back into XNAT. Using XNAT's web services, we can expose that read/write API to other applications on a case by case basis. This will allow qualified users to apply their own analysis methods to the OpenFMRI database and then expose the results via the database. This platform model will give end users a variety of choices in how their data are processed, while providing automated documentation and quality control.

In addition to being hosted directly by the openfmri.org web site, the shared dataset is also available via the INCF Dataspace (http://www.incf.org/resources/data-space), which is a federated data sharing environment based on the iRODS data management system.

## 6. Current status and future plans

The OpenFMRI database currently contains 18 full datasets from seven different laboratories, with a total of 347 unique scanning sessions, all of which are available for download directly from the web site. The database remains heavily skewed toward datasets from the Poldrack lab, but is increasingly diverse with the addition of new datasets. The site is currently accepting uploads, and has a number of datasets in the process of curation in addition to those currently available for download. As of March 2013, there have been 914 downloads of full datasets from the database, and four publications using data from the OpenfMRI database (Carp, 2012; Pedregosa et al., 2012; Varoquaux et al., 2012; Park et al., 2013).

Development, curation, and further population of the site are currently funded by a set of linked grants from the National Science Foundation. However, as Van Horn and Gazzaniga (2012) point out, it is essential to have a plan for longevity once the initial funding period ends. We are hopeful that national funding agencies will continue to view this project as worth supporting, but cannot rely on this. The Texas Advanced Computing Center has committed to long-term storage and accessibility of the data, but continued operation of the project beyond the funding window will require volunteer curators. Given the increased attention to data management and data sharing by federal funding agencies, it is possible that curation could also be supported by “data managers” funded by grants in participating labs. We will also explore other options for long-term funding such as development of a non-profit organization (similar to Wikipedia).

## 7. Preliminary analyses

To demonstrate the potential utility of mega-analysis on a large task-based fMRI dataset, below we present results from initial analyses of a subset of the current database (as of March 2013). The datasets, tasks, and contrasts included in this analysis are listed in Table 2. Three datasets from the current database were excluded from this analysis, due to small sample size (ds105) or exact replication of tasks and subjects from other datasets (ds006B and ds017B). Most of the datasets include multiple runs, for a total of 479 images from 337 unique subjects for run 1, and 429 images from 317 unique subjects for run 2.

**Table 2:** **List of datasets used in the preliminary analyses below**.

**Dataset #**	**Accession #**	**Task #**	**Task/contrast description**	**References**
1	ds001	1	Balloon analog risk task: Parametric pump effect vs. control	Schonberg et al., 2012
2	ds002	1	Classification learning task: Task vs. baseline	Aron et al., 2006
3	ds002	2	Classification learning task: Feedback vs. baseline	Aron et al., 2006
4	ds002	3	Classification decision: Task vs. baseline	Aron et al., 2006
5	ds003	1	Rhyme judgment: Task vs. baseline	Xue and Poldrack, 2007
6	ds005	1	Mixed-gambles task: Parametric gain response	Tom et al., 2007
7	ds006A	1	Mirror reading task: Mirror-reversed vs. plain items	Jimura et al., in preparation
8	ds007	1	Stop signal task: Letter classification vs. baseline	Xue et al., 2008
9	ds007	2	Stop signal task: Letter naming vs. baseline	Xue et al., 2008
10	ds007	3	Stop signal task: Pseudoword naming vs. baseline	Xue et al., 2008
11	ds008	1	Stop signal task: Successful stop vs. baseline	Aron et al., 2007
12	ds008	2	Conditional stop signal task: Successful stop vs. baseline	Aron et al., 2007
13	ds011	1	Tone-counting task: Task vs. baseline	Foerde et al., 2006
14	ds011	2	Single-task classification learning: Task vs. baseline	Foerde et al., 2006
15	ds011	3	Dual-task classification learning: Task vs. baseline	Foerde et al., 2006
16	ds011	4	Classification decision: Task vs. baseline	Foerde et al., 2006
17	ds017A	2	Conditional stop signal task: Go-critical vs baseline	Rizk-Jackson et al., unpublished
18	ds051	1	Abstract-concrete task: novel vs. repeated words	Alvarez and Poldrack, unpublished
19	ds052	1	Classification learning task: Positive feedback vs. baseline	Poldrack et al., 2001
20	ds052	2	Classification reversal learning task: Negative feedback vs. baseline	Poldrack et al., 2001
21	ds101	1	Simon task: incorrect vs. correct	Kelley and Milham, unpublished
22	ds102	1	Flanker task: incongruent vs. congruent	Kelly et al., 2008
23	ds107	1	One-back task: words vs. consonants	Duncan et al., 2009
24	ds108	1	Emotion regulation task: Regulate-negative vs. Look-negative	Wager et al., 2008
25	ds109	1	False belief task: False belief story vs. false picture story	Moran et al., 2012
26	ds110	1	Incidental memory encoding task with cueing: Valid cue high confidence hits vs. misses	Uncapher et al., 2011

Accession and task numbers refer to the specific descriptors used in the database.

All analyses reported below were performed on spatially normalized Z statistic maps obtained for the contrast of interest using the processing stream described in Table 1. The data used to generate all for the foregoing analyses and figures are available from the OpenFMRI web site at http://openfmri.org/dataset/ds900001, and the code used to perform all analyses is available at http://github.com/poldrack/openfmri. Thus, anyone should be able to run these same analyses on their own system in order to reproduce the results reported here.

### 7.1. ICA analysis

**Figure 2:**
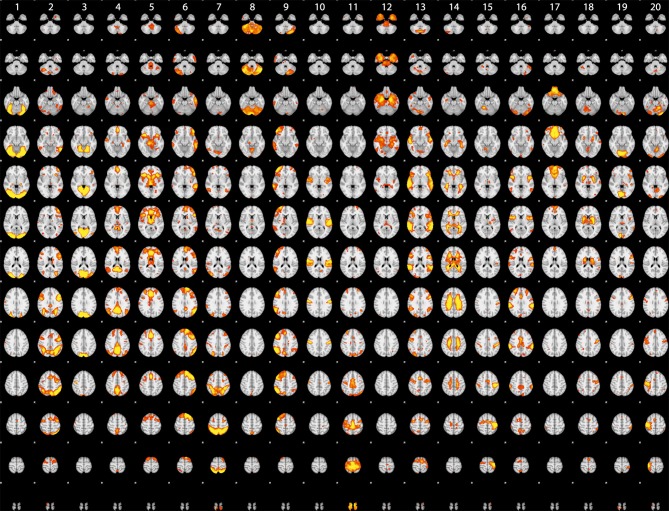
**Rendered maps of the voxels with significant loadings on the 20 ICA components identified statistical images for the datasets listed in Table 1**. Each column displays the loading for a single component; voxels shown in red had positive loading for that component.

Although the statistical maps obtained in the analyses described above include more than 200,000 voxels, a significant amount of information is carried in the coordinated activity of a much smaller number of large-scale neural systems, which can be identified using dimensionality reduction methods such as independent components analysis (ICA). To characterize the large-scale networks that emerged across the different tasks in the dataset, statistical (Z) images for each contrast/task/dataset were submitted to ICA using the FSL melodic tool (Beckmann and Smith, 2004) after spatial smoothing (6mm FWHM). Based on similar recent analyses (Smith et al., 2009; Congdon et al., 2010), we first specified 20 components in order to identify a small set of large-scale networks. The components identified in this analysis are shown in Figure 2. A number of these components reflected the basic sensorimotor aspects of the tasks, including visual regions (components 1, 3, and 19), auditory regions (component 10), and motor regions (components 15 and 20). Others reflected higher-order networks, including fronto-parietal (component 2) and cingulo-opercular (component 5) control networks identified by Dosenbach et al. (2010) and the left-hemisphere language network (component 6). In addition, there were components reflecting the “default-mode” network generally identified during the resting state (component 4) as well as one component reflecting coherent white matter signal (component 14). These results are highly consistent with the results of Smith et al. (2009), which were based on meta-analytic maps from the BrainMap database.

### 7.2. Classification analysis

Previous work (using some of the same data analyzed here) has shown that it is possible to classify which task a subject is performing using a classifier that was trained on other individuals performing a broad range of tasks (Poldrack et al., 2009). We performed a set of similar analyses in order to examine the replicability of those analyses in a dataset that overlapped partially with those used by Poldrack et al. (2009) but using different dimensionality reduction and classification methods. We trained a classifier to identify the task being performed by each subject out of 26 possible tasks. To reduce the dimensionality of the dataset, the whole-brain data from run 1 were regressed against the spatial ICA components obtained from the run 2 data (in order to maintain strict separation of training and test data). ICA was estimated using several different dimensionality levels, in order to examine the relation between classification accuracy and degree of dimensionality reduction; in each case, the loadings on each component (ranging from 2 to 200 components) were used as features in the classification. Twenty-six-way classification was performed using three methods: Linear support vector machine (SVM) (implemented in Liblinear: Fan et al., 2008), non-linear support vector machine (with radial basis kernel, implemented in LibSVM: Chang and Lin, 2011) and regularized logistic regression (LR) with an L2 penalty (implemented in the scikit-learn package: http://scikit-learn.sourceforge.net/). Classifier parameters (cost parameter for SVMs, gamma for non-linear SVM, and penalty parameter for LR) were optimized using the run 2 data, ensuring no crosstalk between parameter identification and classification testing. Classification accuracy was assessed on the run 1 data using leave-one-out cross validation, and an empirical null distribution was obtained by performing the classification 1000 times using randomly permuted labels.

**Figure 3:**
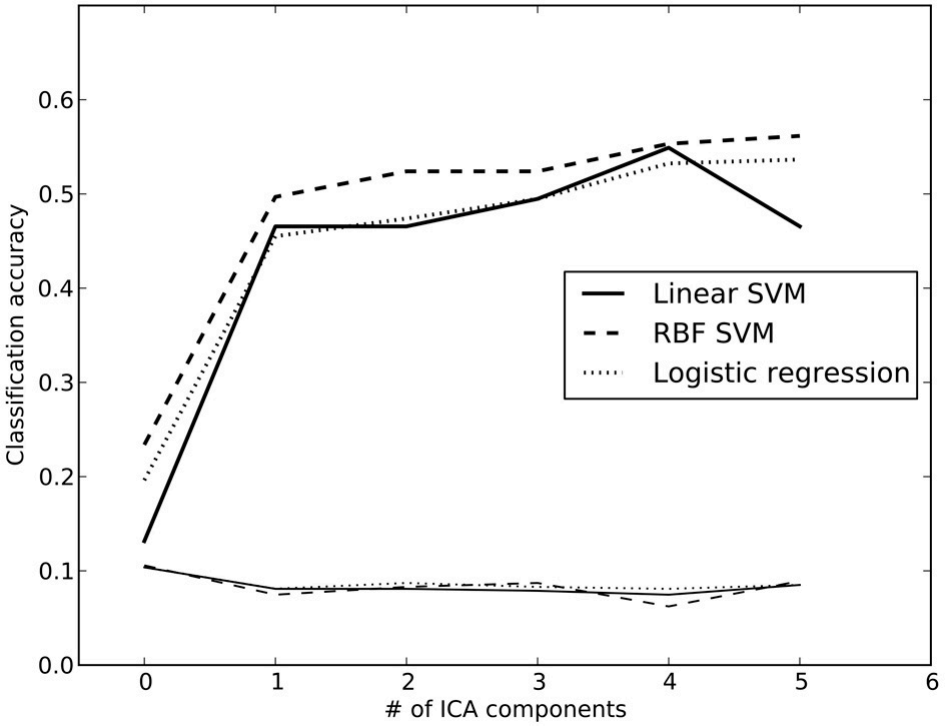
**Classification accuracy using reduced-dimension data, as a function of the number of ICA components in the dimensionality reduction step**. Dimensionality reduction was first performed on an independent set of data (from run 2 for each subject), and the data from run 1 were then projected onto those components. Reported accuracy (thick lines) reflects average accuracy of task classification across all data points, from a total of 25 possible labels. The thin lines at the bottom reflect the empirically derived 95% cutoff for the null hypothesis of chance accuracy, obtained by performing the same classification 100 times with randomized labels, and taking the 95th largest value. RBF, radial basis function; SVM, support vector machine.

Accuracy for the 26-way classification for each method across all dimensionality levels is shown in Figure 3. Accuracy rose incrementally as the number of components was increased from 2 to 100 and then remained relatively stable (around 50%) after 100. Accuracy was quite similar for the two linear classifiers, and only slightly higher for the non-linear SVM. All classification values were substantially greater than chance. Thus, highly significant classification was possible across subjects on a range of tasks, even after very substantial dimensionality reduction, consistent with the findings of Poldrack et al. (2009). It should be noted that because some subjects contributed data to multiple datasets in the classification analysis, not all data points were independent; this likely led to reduced classification accuracy due to confusions caused by subject-specific rather than task-specific patterns. Analysis of classifier confusion matrices showed that discriminability between tasks was compromised in some cases where the subjects overlapped, but in many cases also reflected overlap in task content across different datasets. A further analysis of the effects of non-independence is beyond the scope of the present paper but will be explored in future publications.

We also examined whole-brain classification of Z-statistic maps with the same dataset using a linear support vector machine (SVM) classifier with default cost value (*C* = 1) and eight-fold balanced cross-validation. Voxels with missing data for more than 3 subjects were excluded, leaving a total of 174,264 voxels. Classification accuracy for this analysis was moderate (mean = 48.8%; 95th percentile of empirical null distribution = 7.7%). The decreased accuracy compared to the previously reported task-classification results (Poldrack et al., 2009) likely reflects the fact that the present analysis included more fine-grained contrasts, as well as including a larger number of heavily overlapping tasks. It is interesting that classification performance was not appreciably greater for whole-brain analysis than for the model using ICA components, suggesting that most of the differentiation between tasks is being carried by differences across large-scale networks.

Finally, we examined whether it was possible to identify individual subjects based on their statistical maps. A one-vs.-all multi-class linear SVM was trained to classify each subject into a separate class using the default cost parameter (*C* = 1.0); for some subjects data were available for multiple tasks, whereas for others there was only a single training instance. To ensure that the classification was not driven by missing data, only voxels with non-zero values for all subjects were included in the analysis, leaving a total of 152,704 voxels. Generalization was tested on the data from run 2, which was available for 317 of the 337 subjects. Classification accuracy of 66.9% was achieved; with random subject relabeling, the mean classifier accuracy was 0.3% and the 95th percentile of the null distribution was 0.9%, showing that it was possible to identify individual subjects using their statistical maps from different scanning runs with highly significant accuracy. This finding is consistent with previous arguments regarding the importance of stable individual differences in neuroimaging data (e.g., Miller et al., 2009).

### 7.3. Cluster analysis

**Figure 4:**
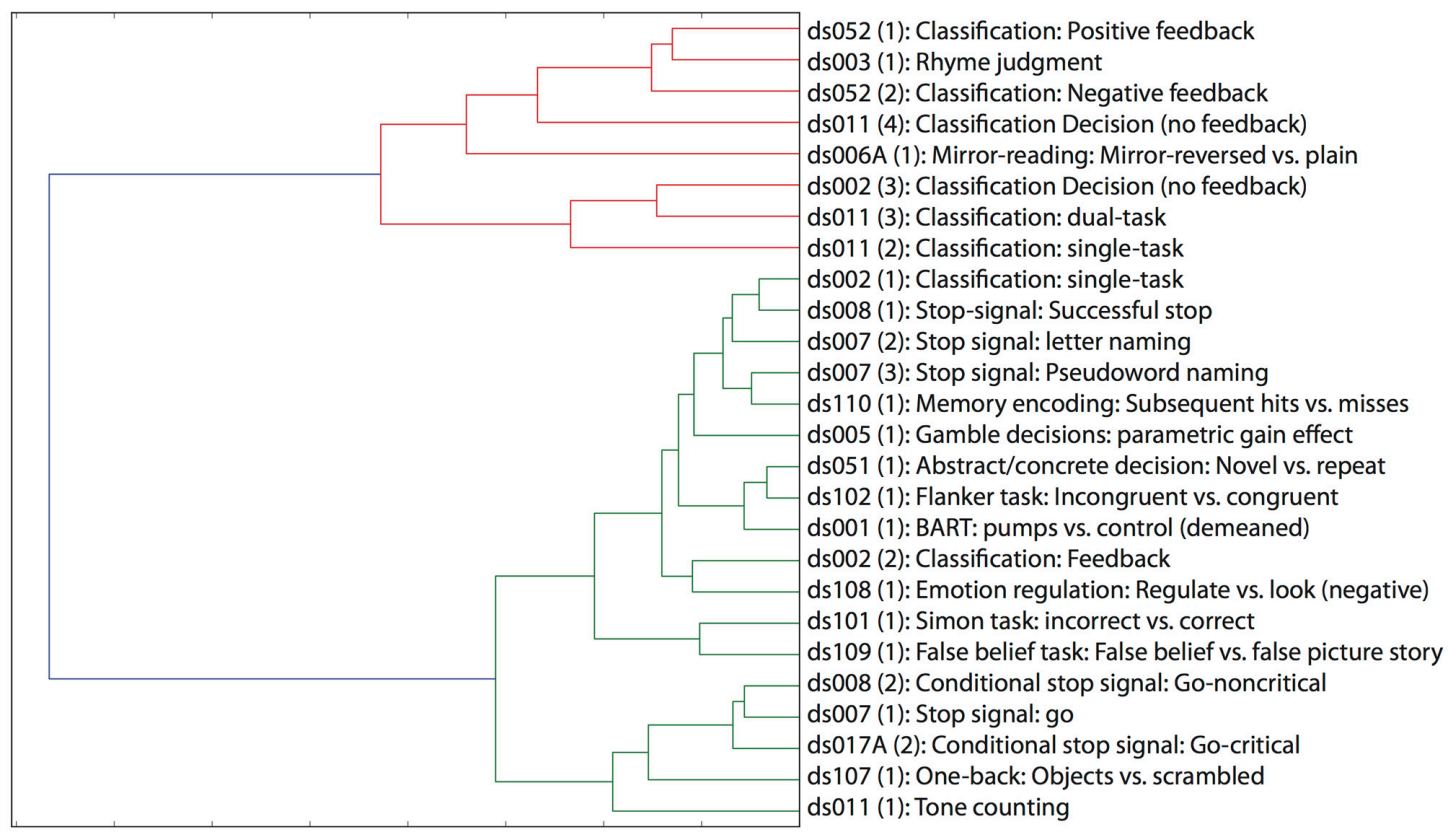
**Hierarchical clustering of statistical maps across tasks, after projection into the 20-dimensional ICA space depicted in Figure 2**.

One of the great advantages of large datasets like those in the OpenFMRI database is the ability to examine the large-scale multidimensional neural space that characterizes different cognitive tasks. In order to examine this, we performed a hierarchical clustering analysis on whole-brain statistical maps after projection into the 20-dimension ICA space depicted in Figure 2 and averaging across subjects within each task; clustering was performed using Ward's method with a Euclidean distance metric as implemented in scikit-learn. The resulting dendrogram is shown in Figure 4, and shows that there is a noisy but surprisingly consistent similarity in the neural activity patterns between tasks that engage common processes [as found previously by Poldrack et al. (2009)]. In several cases, maps from similar tasks within the same dataset were clustered together (e.g., the pseudoword naming and letter naming conditions from ds007, both of which come from the same subjects), whereas in other cases, the same task from different datasets were clustered together; particularly striking is the fact that the classification decision and classification learning datasets from multiple studies are clustered together, even though they were collected on very different versions of the tasks and different scanner platforms. These results highlight the degree to which different tasks exist within a larger similarity space of neural activity, which could potentially provide insights into the latent neurocognitive bases of mental functions.

## 8. Conclusion

Data sharing has revolutionized other areas of biomedical science, and we believe that it has the potential to do the same for cognitive neuroscience. The OpenfMRI data sharing project has developed the infrastructure for the sharing of task-based fMRI datasets, and has begun making datasets openly available online. We are optimistic that this project will help encourage widespread voluntary data sharing by providing a powerful resource that makes sharing as straightforward as possible. Preliminary analyses of the database have confirmed the ability to classify mental states across individuals, as well as demonstrating the novel ability to classify the identity of individual subjects from their fMRI patterns. In addition, multivariate analyses provide new glimpses into the multidimensional relations between mental function and brain function. We foresee many additional insights arising from these data as the database grows and other novel analysis methods are applied to the data.

### Conflict of interest statement

The authors declare that the research was conducted in the absence of any commercial or financial relationships that could be construed as a potential conflict of interest.
